# Supplementary material for: Low-frequency variation near common germline susceptibility loci are associated with risk of Ewing sarcoma
Source: PLoS One. 2020 Sep 3;15(9):e0237792. doi: 10.1371/journal.pone.0237792 (PMC7470401; doi:10.1371/journal.pone.0237792)

**S1 Fig. LDassoc regional association plots for identified rare and low-frequency variant associations with EwS susceptibility.** Plots are for rs78119607 (**A**), rs112837127 (**B**), and rs2296730 (**C**).

(**A**)


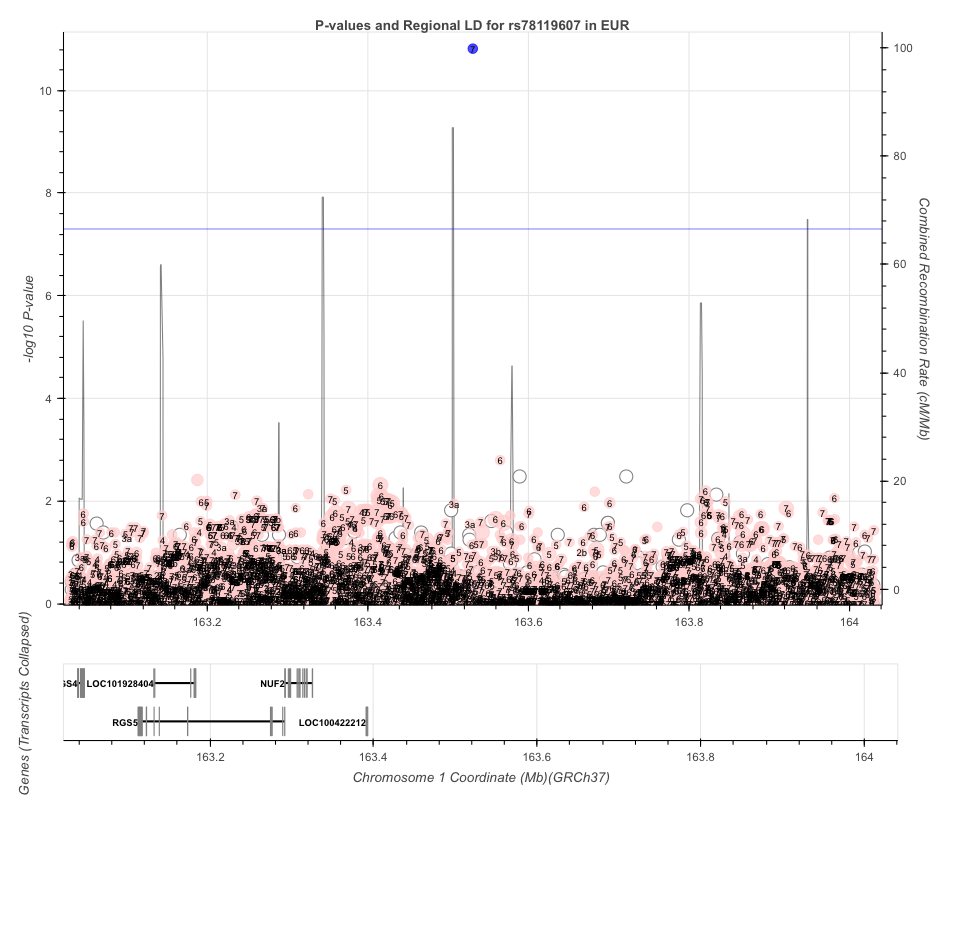


(**B**)


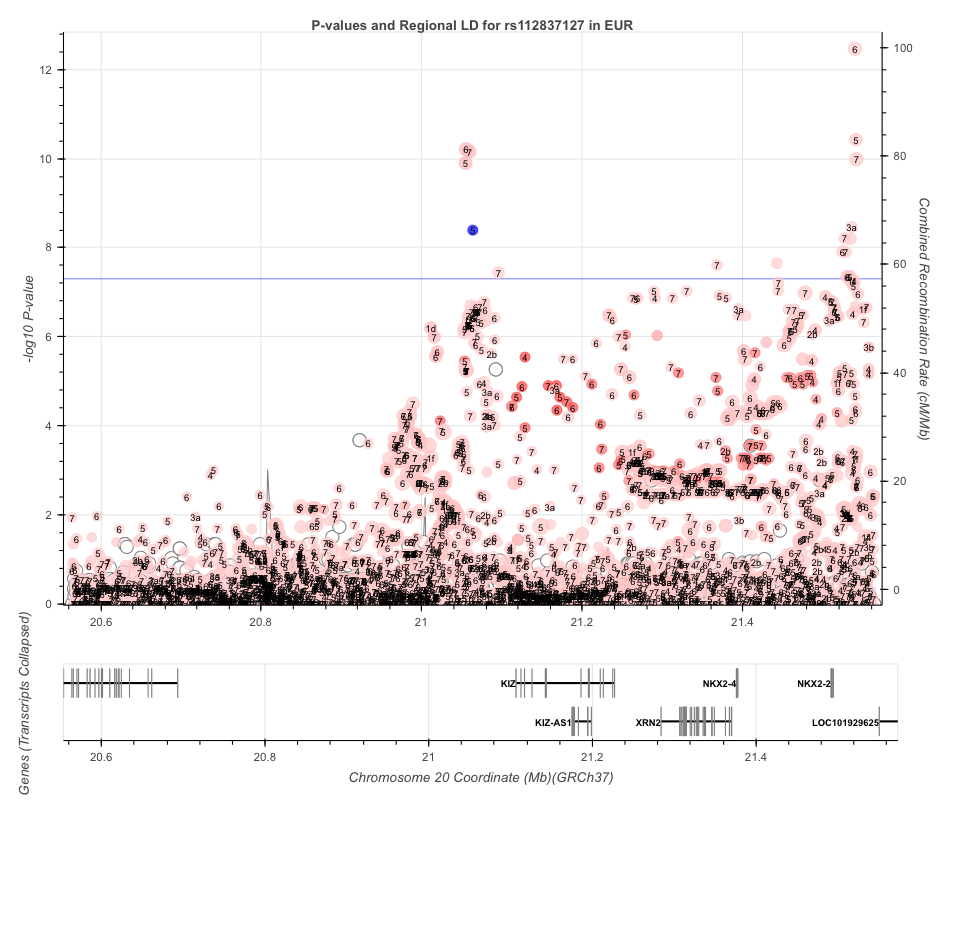


(**C**)


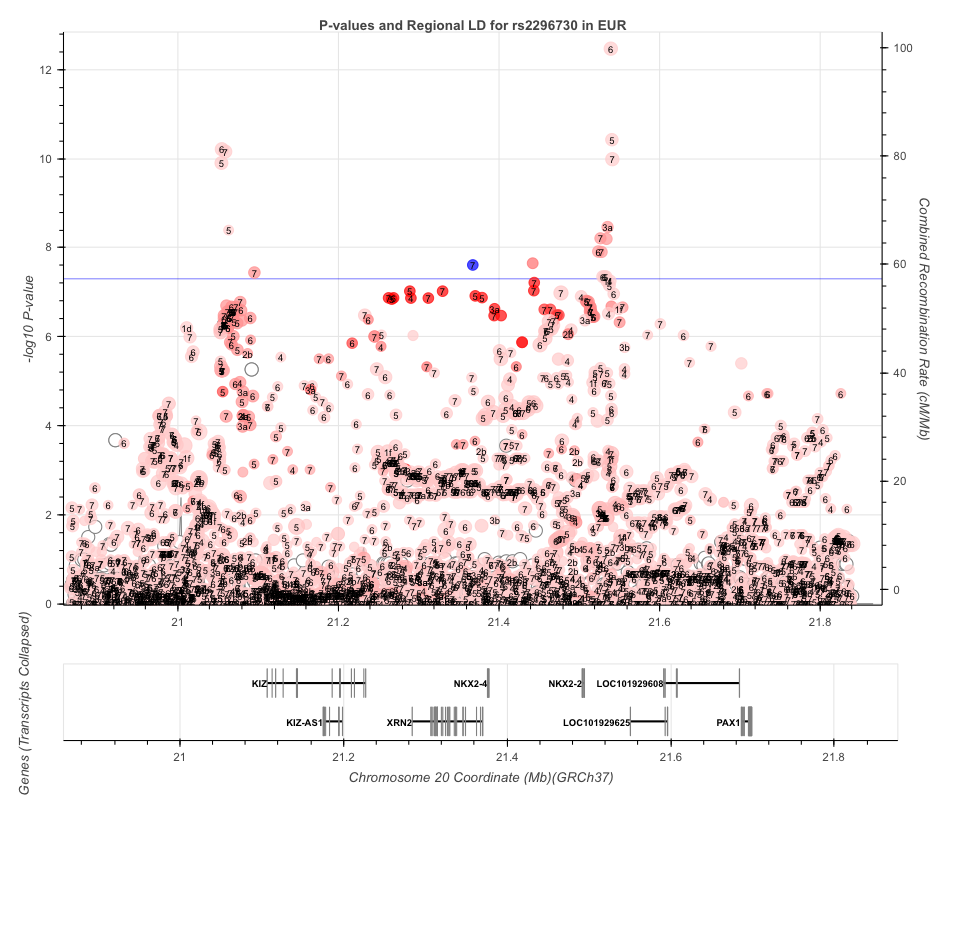

Supplement: S1 Fig — Plots are for rs78119607 (A), rs112837127 (B), and rs2296730 (C). (DOCX) [file pone.0237792.s001.docx]
