# Supplementary figures and images for: Low-frequency variation near common germline susceptibility loci are associated with risk of Ewing sarcoma
Source: PLoS One. 2020 Sep 3;15(9):e0237792. doi: 10.1371/journal.pone.0237792 (PMC7470401; doi:10.1371/journal.pone.0237792)

**S2 Fig. Validation results of EwS associated rare and low-frequency variants by TaqMan assays.**


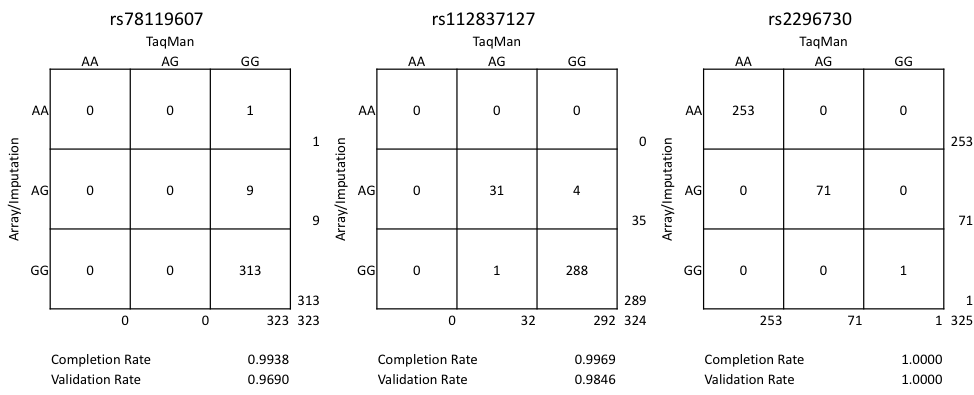

Supplement: S2 Fig — (DOCX) [file pone.0237792.s002.docx]
