# Supplementary material for: Low-frequency variation near common germline susceptibility loci are associated with risk of Ewing sarcoma
Source: PLoS One. 2020 Sep 3;15(9):e0237792. doi: 10.1371/journal.pone.0237792 (PMC7470401; doi:10.1371/journal.pone.0237792)

**S3 Fig. Linkage disequilibrium between the common variant rs6106336 and the two identified low frequency variants** (A) rs112837127 and (B) rs2296730 using LDpair and all European 1,000 Genomes populations as a reference.

(**A**)


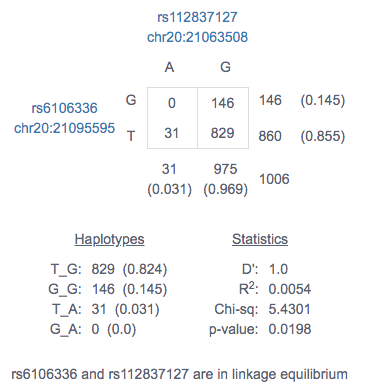


(**B**)


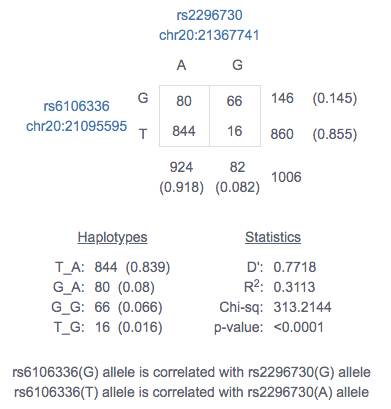

Supplement: S3 Fig — Linkage disequilibrium between the common variant rs6106336 and the two identified low frequency variants (A) rs112837127 and (B) rs2296730 using LDpair and all European 1,000 Genomes populations as a reference. (DOCX) [file pone.0237792.s003.docx]
