# Supplementary material for: Low-frequency variation near common germline susceptibility loci are associated with risk of Ewing sarcoma
Source: PLoS One. 2020 Sep 3;15(9):e0237792. doi: 10.1371/journal.pone.0237792 (PMC7470401; doi:10.1371/journal.pone.0237792)
Supplement: S1 Table — (DOCX) [file pone.0237792.s004.docx]

**S1 Table. Imputation quality scores for each associated low-frequency or rare variant by EwS imputation set.**

|  | **Childhood Cancer Survivor Study** | | **Institute Curie + NCI** | | **Postel-Vinay et al.** | |
| --- | --- | --- | --- | --- | --- | --- |
|  | **Info** | **Certainty** | **Info** | **Certainty** | **Info** | **Certainty** |
| rs78119607 | 1 | 1 | 0.433 | 0.996 | 0.609 | 0.997 |
| rs112837127 | 1 | 1 | 0.843 | 0.989 | 0.804 | 0.987 |
| rs2296730 | 1 | 1 | 0.984 | 0.998 | 1 | 1 |
