# Supplementary material for: Low-frequency variation near common germline susceptibility loci are associated with risk of Ewing sarcoma
Source: PLoS One. 2020 Sep 3;15(9):e0237792. doi: 10.1371/journal.pone.0237792 (PMC7470401; doi:10.1371/journal.pone.0237792)
Supplement: S2 Table — (DOCX) [file pone.0237792.s005.docx]

**S2 Table. Distribution of alleles across three EwS study populations.**

|  | **Childhood Cancer Survivor Study** | | **Institute Curie + NCI** | | **Postel-Vinay et al.** | |
| --- | --- | --- | --- | --- | --- | --- |
|  | **Ref**  **N (%)** | **Alt**  **N (%)** | **Ref**  **N (%)** | **Alt**  **N (%)** | **Ref**  **N (%)** | **Alt**  **N (%)** |
| rs78119607 | 1019 (99.9) | 1 (0.09) | 943 (97.0) | 29 (2.98) | 2161 (99.7) | 5 (0.23) |
| rs112837127 | 999 (97.9) | 21 (2.05) | 951 (97.8) | 21 (2.16) | 2112 (97.5) | 54 (2.49) |
| rs2296730 | 960 (94.1) | 60 (5.88) | 923 (94.9) | 49 (5.04) | 2019 (93.2) | 147 (6.78) |
